# Supplementary material for: Are carbon emissions trading and green financial instruments synergistic? -Comprehensive quantitative research based on content analysis
Source: PLoS One. 2024 Mar 7;19(3):e0298601. doi: 10.1371/journal.pone.0298601 (PMC10919601; doi:10.1371/journal.pone.0298601)
Supplement: S1 Data — (ZIP) [file pone.0298601.s001.zip › Data/地级市Z_F工作报告.zip!/╔╜╬≈/╜·│╟╩╨/└┤╫╘╣1⁄2╓┌║┼í╛┬φ┐╦╩2╛▌═°í┐_2003.docx]

各位代表：

现在，我代表市人民政府向大会作政府工作报告，请予审议，并请各位政协委员和其他列席人员提出意见。

　　一、关于2002年政府工作的回顾

　　过去的一年，在市委的正确领导下，市政府团结和带领全市人民，紧紧围绕“一争两转五突破”的工作部署，奋力进取，争先发展，经济建设和各项社会事业取得了新的成绩。

　　(一)主要经济指标保持快速增长。全市国内生产总值完成172亿元，比上年增长11．5％；规模以上工业增加值完成51．3亿元，比上年增长工6％；乡镇企业增加值完成54．7亿元，比上年增长11．1％；财政总收入完成22．29亿元，比上年增长31．5％。其中，一般预算收入完成8，07亿元，比上年增长26．7％；城镇居民人均可支配收入达到6107元，比上年增长13．6％；农民人均纯收入扭转徘徊局面，达到2707元，比上年增长4．4％；社会消费品零售总额完成50，04亿元，比上年增长8．6％；外贸进出口总额完成2002万美元，比上年增长54．3％。

　　(二)经济结构调整成效显著。农业结构调整势头良好，特色农产品生产基地稳步推进，农业产业化经营格局初具雏形，退耕还林完成17．5万亩。传统产业提升和新兴产业培育步伐加快。骨干煤矿建设进展顺利，累计建成100万吨矿井3座，60万吨矿井2座，30万吨矿井25座。积极实施煤转肥、煤转电战略，尿素生产能力达到43万吨，阳电一期工程6台机组210万千瓦装机能力全部投．入运营。冶炼、铸造业坚持联动互调，有6座179立方米以上高炉建成投产，泫氏球墨铸管等一批中高档铸造项目建设进展顺利。技术改造力度进一步加大，全市安排的59项技改项目有45项投产，其中10项重点技改项目有8项投产。以太行山水风情游为标志，全力打造旅游品牌，为经济结构调整增添了新的亮点。民营经济销售收入同比增长13％，上交税金同比增长26％。

　　(三)城市建设和管理取得新的成绩。以改善人居环境、提升城市品位为中心，在继续抓好城市道路建设的同时，加大了城市绿化和文化、体育设施建设力度。“三路两桥”全部贯通。环城绿色通道、公共绿地和拆墙透绿三项绿化工程新增绿地14l万平方米，城市绿化覆盖率和绿地率分别达到32％和24％，人均公共绿地达到6平方米。凤鸣中学、凤西广场、博物馆、游泳馆、体育训练馆投入使用，多功能综合馆开工建设。城市环境综合整治取得阶段性成效，城市管理上了新的台阶。

　　(四)改革开放迈出较大步伐。以明晰产权为核心，积极推进企业改革，阳城制丝有限公司等5家国有困难企业的改革脱困工作取得实质性进展，乡镇企业采取拍卖、租赁、股份制等多种方式加大了改制力度，民营企业按照现代企业制度开始实施公司制改造。坚持“引进来”和“走出去”相结合，组团参加了厦洽会、乌洽会、深圳高交会等全国性的招商引资活动，全市自营进出口企业达到27家，进出口总额增幅位居全省第三。经济技术开发区建设有了实质性进展，科工贸总收入完成7．3亿元，财政总收入完成1627万元，一个新的经济板块正在崛起。

　　(五)经济发展环境进一步优化。投资硬环境继续改善，城乡电网改造基本完成，晋焦高速公路全线通车，晋长高速公路正式开工，村村通油路全面启动，张峰水库项目已经国家批准立项，三年饮水解困任务基本完成。行政审批制度改革步伐加快，分两批共取消、下放、转移审批事项366项。社会保障体系进一步完善，下岗职工按时足额领到了基本生活费，企业离退休职工养老金按时足额发放率和社会化发放率均达100％，失业保险覆盖面达到95％，医疗保险覆盖面达到70％，城市低保基本实现应保尽保，农村低保稳步推进。整顿和规范市场经济秩序工作进一步加强。认真落实责任，切实加强监管，安全生产形势明显好转。

　　(六)科技、教育等各项社会事业全面发展。全市共推广新技术102项，引进专利技术52项，开发新产品36项，科技进步对经济增长的贡献率稳步提高。完成中小学危房改造283所，中小学布局调整取得阶段性成果。市歌舞团和少儿艺术团完成组建，30集电视连续剧《长平之战》开机拍摄。卫生预防和医疗体系进一步完善，“百万农民健康行动”成效显著，成为全国农村卫生工作10个示范试点地市之一。省运会成绩位次前移，群众体育富有成效。计划生育、国土资源管理和环境保护工作继续加强。人防工作成绩显著。广播、电视、文物、民政、民族、宗教、外事、侨务、档案、气象、地震、老龄、残疾人等各项工作也都有了新的变化。

　　(七)社会主义精神文明和民主法制建设得到加强。“三个代表”学习教育活动深入开展，思想道德建设和精神文明创建活动卓有成效。市政府及时向市人大及其常委会报告工作，经常听取市政协、各民主党派、无党派人士和专家学者的意见，努力拓宽同人民群众的联系渠道，办理人大代表建议183件、政协委员提案27l件。认真强化社会治安综合治理，深入开展“严打”斗争，积极化解各种矛盾，维护了社会大局稳定。注重从源头上、制度上、机制上预防和治理腐败，廉政建设和纠风工作取得新的成效。

　　各位代表，我市2002年经济建设和社会事业取得的成绩来之不易。这是中共晋城市委正确领导的结果，是市人大、市政协监督支持的结果，是全市人民团结奋斗的结果。在此，我代表市人民政府向奋战在全市各条战线的工人、农民和知识分子，向为我市经济社会发展做出贡献的驻市部队、武警官兵、驻地企业和单位，向大力支持政府工作的人大代表、政协委员和各界人士，向所有关心支持晋城发展的同志们、朋友们致以崇高的敬意和衷心的感谢!

　　在肯定成绩的同时，我们也清醒地看到，前进中还存在不少困难和问题。主要是：产业单一、产品初级的资源型经济特征仍然比较突出，保持经济稳定快速增长的基础还不牢固；农村富余劳动力转移困难，农民增收缓慢；城镇就业渠道不畅，社会保障压力较大；环境保护和生态建设的任务还相当艰巨；资本、产权、技术、人才等市场体系发育不够；城市规划滞后，城市管理的长效机制不健全，经营城市理念还没有真正确立；政府职能转变还不到位，官僚主义、形式主义在一些地方和部门还时有发生。对这些困难和问题，我们必须高度重视，采取有效措施，认真加以解决。

　　二、关于2003年政府工作的总体要求和主要任务

　　各位代表，党的十六大提出了全面建设小康社会的奋斗目标和行动纲领，为开创中国特色社会主义事业新局面指明了方向。市委、市政府在全面分析形势的基础上，明确提出到2007年国内生产总值由2000年的145亿元增加到290亿元，年均递增10．4％，实现新世纪、新阶段的第一个翻番，进而为提前实现第二个翻番奠定坚实的基础。因此，我们必须树立强烈的挑战意识、机遇意识、创新意识和发展意识，努力做到发展要有新思路，改革要有新突破，开放要有新局面，各项工作要有新举措，以跨越式发展的实际行动，迈出全面建设小康社会，的坚实步伐。

　　全面建设小康社会，关键在于开好局、起好步。因此，做好今年的政府工作意义十分重大。今年我市政府工作的总体要求是：紧紧围绕全面建设小康社会的总目标，以邓小平理论和“三个代表”重要思想为指导，认真贯彻落实党的十六大和全国“两会”精神，按照省委、省政府对我市争先发展的要求，继续贯彻市委“一争两转五突破”的工作部署，突出争先发展这个主题，扭住经济结构调整这条主线，明确“四市”建设目标(经济强市、园林城市、一流文明城市、优秀旅游城市)，加快“四化”发展步伐(新型工业化、农业产业化、城市化、信息化)，确保四个快速增长(国内生产总值、财政总收入、城镇居民人均可支配收入、农民人均纯收入)，促进全市国民经济持续快速健康发展和社会全面进步。

　　今年我市经济和社会发展的主要预期目标为：国内生产总值187亿元，增长11％；财政总收入25．9亿元，增长16．1％。其中，一般预算收入8．79亿元，增长工2，3％；城镇居民人均可支配收入6720元，增长10％；农民人均纯收入2900元，增长7％；城镇登记失业率控制在4%以内；人口自然增长率控制在7.1‰以内。以上预期目标是综合考虑各方面的有利条件和制约因素而确定的，是符合争先发展要求和我市实际的，是切实可行的。应该看到，完成今年的任务，我们面临许多有利条件：党的十六大提出全面建设小康社会的宏伟目标，极大地激发了全市人民加快发展的愿望和信心；中央继续实行积极的财政政策和稳健的货币政策，有利于我们扩大投资和消费需求；东部加速产业升级、西部实施大开发的宏观背景，有利于我们引进资金、项目、技术和人才；特别是经过近些年的努力，我市综合经济实力进一步壮大，为争先发展奠定了较好的物质基础。只要我们众志成城，同心同德，只要我们集中民智，发挥民力，把党的十六大之后广大干部群众焕发出来的干劲、智慧和积极性，引导到全面建设小康社会的伟大进军中来，我们就一定能够克服前进中的困难，圆满实现今年的各项目标，并力争发展得更快更好。对此，我们充满必胜的信心。为了圆满实现今年经济社会发展的预期目标，我们必须着力

做好以下七个方面的工作：

　　(一)加速产业结构的战略性调整，努力推进跨越式发展提升优化产业结构，提高经济增长的质量和效益，是争先发展的本质要求。我们必须清醒地看到，我市丰富的煤铁资源既养育了

我们，同时也束缚了我们。近几年我市的产业结构调整虽然取得了明显成效，但目前资源型传统产业增加值占第二产业增加值的比重仍高达80％以上，提供的财政收入占财政总收入的比重也高达55％左右。我们必须充分正视这一现实，紧紧抓住当前煤炭市场看好的大好时机，站在晋城长远发展的战略高度，坚定不移地把经济结构调整抓紧抓实。

　　要牢固树立工业立市的理念。加速工业化进程，是实现现代化的必由之路。我市虽然建立起了初步的工业体系，但加工制造业水平较低，目前还处于工业化中期的初始阶段。我们必须按照走新型工业化道路的要求，叫响叫亮工业立市，紧紧围绕煤炭、电力、化肥、冶铸、建材、丝麻、饮品等七大产业建设，以信息化带动工业化，以工业化促进信息化，推动全市工业结构优化升级，走出一条科技含量高、经济效益好、资源消耗低、环境污染少、人力资源优势得到充分发挥的新型工业化道路。

　　要坚持用高新技术和先进适用技术改造提升传统产业。煤炭产业要坚持总量控制、优化结构的原则，继续实施“关小改中建大”战略，大力提高矿井集中度和装备水平，年内要关闭小煤矿54座，改造和建设6座100万吨矿井、2座60万吨矿井、12座30万吨矿井，累计建成9座100万吨矿井、4座60万吨矿井、37座30万吨矿井，骨干矿井产量达到2200万吨，占到煤炭总产量的50％以上。同时，抓好采煤方式改革、筛分系统改造和煤炭加工转化工作，块炭率达到55％以上，加工转化率达到25％以上。冶铸产业要坚持“关小建大抓示范”，年内要关闭100立方米以下高炉100座，建成225立方米高炉1座，318立方米高炉2座，累计建成179立方米以上高炉9座。全年生铁产量达到230万吨。要抓好5条中高档铸造生产线的达产达效，开工建设市经济技术开发区精密铸造工业园区，积极发展高科技含量、高附加值的精密铸造业，全年铸件产量达到120万吨，其中中高档铸件产量达到30万吨以上，为把我市建设成为全国重要的铸造基地之一打好基础。化肥产业要完成晋城煤化工公司和天脊集团晋城化工公司两个“1830”项目建设，新增尿素生产能力60万吨，确保年底达到103万吨生产能力，力争到2004年底全面实现国家确定的200万吨优质无烟煤化肥基地建设任务。 电力产业要积极推动阳电二期120万千瓦机组和鲁能集团煤电铝项目尽早开工。建材、丝麻、饮料等产业也都要加快技术进步，培植新的优势。同时，要有选择、有重点地努力培育发展新型材料、精细化工、生物制药等高新技术产业，形成新的经济增长点。

　　要继续加大技术改造和资产重组力度。今年共安排市级技改项目54项，总投资28．3亿元，其中重点技改项目15项，总投资22．8亿元，全部项目达产达效后可新增产值43．3亿元，新增利税7．8亿元，其中今年新增产值1．8亿元，新增利税0．4亿元。要紧紧围绕主导产业建设，积极实施资本运营，大力推进资产重组，着力培育和壮大一批主业突出、核心竞争力强的大公司和大集团，进一步提高工业集中度和规模经营度。每个县(市、区)都要尽快扶持发展1—2个产值10亿元以上的骨干企业，真正构筑起晋城工业经济的台柱子。

　　要全力抓好基础设施建设。今年共安排市级重点工程10项，其中，晋长、晋济洛两条高速公路分别在明年和后年建成，可新增通车里程92公里，使全市高速公路通车里程达到160公里，跨入全省先进行列。晋陵一级公路也要在明年全线通车，同时要积极抓好晋侯高速公路的前期工作。这些交通项目的开工建设，将大大改善我市的公路交通条件，形成四通八达、快速便捷的高速交通网络，为充分发挥区位优势，立足太行，逐鹿中原打开黄金通道。张峰水库上半年要开工建设，这对解决我市水资源严重短缺问题，改善生态环境，保障经济社会可持续发展，具有决定性意义。大宁煤矿、晋城煤化工公司“1830”项目、市综合馆、市体育场、市图书馆、市污水处理厂等重点工程，对增强经济发展后劲、提升城市文化品位、改善城市环境也都具有至关重要的作用。对10项重点工程，有关单位和项目法人必须全力以赴，精心组织，确保工期，确保质量，向全市人民交一份满意的答卷。

　　要集中力量培育旅游产业。旅游产业是当今世界最重要的经济产业之一，被誉为“无烟工业”、“朝阳产业”。我市历史悠久，文化灿烂，山川秀美，旅游资源丰富而独特。全市上下必须进一步提高对发展大旅游的认识，强化政府主导，整合旅游资源，完善基础设施，提升文化品位，注重宣传促销，大力开拓以中原为主的客源市场，全力构建以太行山水风情为灵魂，以炎帝文化、长平古战场、皇城相府为内涵，以王莽岭、珏山、舜王坪、蟒河、太行山峡谷为舞台，融自然风光与人文景观为一体的旅游业发展新格局，真正把旅游资源优势转化为经济优势、产业优势。今年全市接待游客要达到220万人次，努力在较短的时间内跨入全国优秀旅游城市行列。

　　(二)紧扣农民增收这个中心，全面发展农业和农村经济

　　全面建设小康社会，重点和难点在农村，核心是增加农民收入。我市80％以上的人口在农村，没有农民、农村的小康，就没有全面的小康。今年我市农民人均纯收入预期目标为同比增长7％，这个目标要确保实现必须付出艰辛的努力。

　　要大力推进农业产业化经营。充分发挥比较优势，推进优势农产品合理布局，加快建设优质杂粮、蔬菜、果品、蚕桑、中药材、畜牧等六大特色产业带，发展特色产业和产品。推动农业标准化生产，建立健全农产品质量标准体系和检测检验体系，积极发展无公害、绿色和有机农产品。加大对农业产业化龙头企业的扶持力度，引导企业采用新技术，开发新产品，扩张原料基地，拉长产业链条。积极扶持发展农村经纪人队伍和中介组织，提高农民走向市场的组织

化程度。加快农村信息体系、市场体系和科技体系建设，为农业产业化和农民增收提供有效服务。

　　要进一步加快农村城镇化进程。以5个县城和巴公、北留、嘉丰等小城镇为重点，高起点规划，高质量建设，高效能管理，高效益经营。尽快消除不利于小城镇发展的体制和政策障碍，推进户籍制度等配套改革，多渠道、多形式筹措建设资金。以小城镇为依托，突出抓好工业园区建设，引导乡镇企业向工业园区聚集，扩大规模，提升档次，加快二次创业步伐，以工业园区的壮大带动小城镇经济的繁荣和农民收入的增加。完善小城镇功能，大力发展现代服务业，增强吸纳农村富余劳动力的能力，促进农民向非农产业和小城镇转移。

　　要加大农村基础设施建设力度。积极改善农业生产条件，完成退耕还林20万亩，发展节水灌溉3万亩，治理水土流失1．0万亩，改造中低产田30万亩。注重优化农村发展环境，全面完成农村电网改造，加快小水电发展步伐；打好农村公路建设攻坚战，实现5个乡镇、800个行政村通油路或水泥路；继续实施农村饮水解困工程，新解决6万人的饮水困难。延伸太行农网和信息助农网络，推进农业信息化进程。建设基础地理信息系统，强化国土资源依法保护和有序利用工作。

　　要继续深化农村改革。认真执行《农村土地承包法》，稳定土地承包关系，按照“依法、自愿、有偿”的原则，积极探索土地承包经营权流转机制，发展适度规模经营。要继续抓好减轻农民负担工作，推行村务公开，完善涉农价格、收费公示制。全面启动农村税费改革是农民减负增收的重大举措，必须态度坚决，步子扎实，工作到位，确保落实。要推进农产品流通体制改革，促进农产品销售。加快农村金融体制改革，广泛推开农户小额信贷，解决农民贷款难问题。

　　(三)进一步深化经济体制改革，全方位提高对外开放水平深化改革，扩大开放是推动经济发展永不衰竭妁强大动力。我们必须进一步解放思想，开阔视野，牢固树立以改革促调整、以开放促发展的理念，努力推动改革开放再上新水平。

　　要加大所有制结构调整力度。这是带有根本性并事关长远的重大问题。要坚持毫不动摇地巩固和发展公有制经济，毫不动摇地鼓励、支持和引导非公有制经济发展。要按照建立现代企业制度的要求，继续推进国有企业规范的公司制改革和股份制改造。市属国有企业要在两年内通过两个置换，真正成为自主经营、自负盈亏的独立的市场主体，今年要重点抓好晋钢、太印、气门等特困企业的改革。 乡镇企业要通过买断、租赁、股份合作制等以明晰产权为核心的改革，积极推进民营化。全市非煤乡镇企业的改制工作年内要全部完成，对过去已完成改制的要来一次全面的回头看，切实做到体制创新，规范运作。同时，积极探索建立煤炭资源有偿使用制度，使煤炭企业改制在今年取得实质性进展。

　　必须把民营经济的发展放在突出的战略位置来抓。一定要充分认识民营经济在促进经济增长、扩大就业和繁荣市场等方面的重要作用，把放心、放胆、放手发展民营经济作为强市富民的重大举措，积极为民营经济营造宽松有序的发展环境。在具体工作中，要切实做到三个“一视同仁”：一是在市场主体地位上要一视同仁；二是在市场准入上要一视同仁；三是在资源配置上要一视同仁。通过我们的工作，使民营经济乘风破浪，肩负起更大更重的历史使命，为全市经济增长增添更新更大的活力。

　　要加速推进商贸流通业改革和各项配套改革。以市糖酒副食公司的规范化股份制改革为重点，全面推进商贸企业改革，尽快实现国有资本的全部退出。加快传统商贸业的业态创新，大力发展连锁经营、物流配送等现代流通方式。要继续推进投融资体制改革，确立企业法人投资主体地位。中小企业担保公司要在上半年投入运行，有效缓解中小企业贷款难的问题。金融部门要积极探索有效解决我市存贷差逐年扩大的措施和办法，加大对地方经济的支持力度。要加快建立公共财政框架，做好部门预算，推进依法理财，开展增收节支活动，集中财力办必要的事和重要的事。要完善国有资产监管体系，依法保障国有资产安全和增值。要积极培育资本、产权、土地、劳动力和技术市场，加快形成统一、开放、竞争、有序的现代市场体系。

　　要千方百计提高对外开放水平。目前我市的对外开放度很低，外贸进出口总额占国内生产总值的比重只有4％，必须进一步解放思想，与时俱进，大力实施全方位、大开放战略。要树立“大晋城”观念，立足双赢，注重实效，不求所有，但求所在。凡在晋城这块土地上的企业，不论是中央企业、省属企业、外资企业，我们都要视同晋城的企业；凡到晋城投资创业的客商，不论是哪种所有制，都是我们的纳税人；凡关心和支持晋城发展的有识之士，我们都要真

诚相待，热烈欢迎。

　　要大力推进园区经济的发展。发达地区的成功实践证明，工业园区是一个地区经济加速发展的孵化器和助推器，它的优势在于基础设施可以共享，土地可以集约利用，资源可以优化配置，环境可以得到有效保护。总之，它可以产生“集聚效应”。因此，我们必须精心运筹，科学规划，集中必要的人力、物力、财力，下决心办好市经济技术开发区和各个县(市、区)的工业园区。要采取切实有效的措施，加快基础设施建设，优化发展环境，提高招商引资质量，加

快建设步伐。要引导工业项目向开发区和工业园区集中，以尽快形成群体规模效益。政府有关部门要进一步加大扶持力度，使开发区、工业园区尽快成长壮大为我市经济结构调整的先行区、新兴产业的孵化区和体制创新的示范区，成为吸引项目、资金、技术、人才的政策洼地和经济技术研发的战略高地，成为晋城经济新的增长极。

　　(四)加大城市建设力度，提高城市管理水平城市是一个地区的经济、政治、文化中心。在推进我市城市化进程中，我们必须把中心城市的规划、建设和管理作为“重头戏”来抓，尽快完善功能，改善环境，提升品位，增强辐射和带动能力。

　　要坚定不移地走经营城市之路。采取有效措施，强化国有土地收购储备制度，垄断土地一级市场，搞活土地二级市场，公开拍卖市政设施广告权、公用设施经营权和路桥冠名权，以筹集更多的建设资金。今年，红星街、东城路北段、图书馆、旧城改造和东、西两河治理等重点城建项目要采取统一规划、多元投资、市场运作的方式进行建设，改变城市基础设施单纯靠政府投入的传统模式。

　　城市建设要精心实施“五项工程”，全力办好“十三件实事”。一是“蓝天工程”。要通过关停污染企业、强化扬尘防治、治理机动车尾气、推广清洁燃料、扩大集中供热等措施，下大力改善城市空气质量，一年打基础，两年上台阶，三年见成效，今年务求首战必胜，空气质量二级以上天数达到120天；二是“畅通工程”。重点是拓宽、完善城市外环路，新建、改建影响城市交通的断头路、卡脖路，以西环路、北环路和东西大街拓宽为切入点启动旧城改造，完

善城市路网结构。今年要贯通红星街，续建南环街西段，新建泽州南路、东城路北段、新市东街东段和黄华街南段，新增城市道路9公里，两年内实现城市道路基本畅通；三是“绿化工程”。重点是抓好5条街道大树绿化和2个游园、3片绿地建设，同时加大拆墙透绿、拆违还绿、拆临造绿、见缝插绿力度，全面提升城市绿化水平。全年绿化面积不少于100万平方米，城市绿化覆盖率新增3个百分点，达到35％，绿地率新增3个百分点，达到27％，人均公共绿地新增2平方米，达到8平方米。今年，我市要建成省级园林城市；四是“美化亮化工程”。市区主要街道的沿街建筑物，都要按照统一规划，进行立面装饰和亮化，做到白天一街一景，和谐美观，晚上灯光璀璨，夜色绚丽，充分展示城市建设的新风貌；五是“便民工程”。重点是新建4个蔬菜网点、5个水冲式公厕和5处垃圾中转站，特别是要下决心对群众反映强烈的东、西两河进行治理，今年迈出实质性步伐。围绕“五项工程”建设，我们要全力办好与人民群众生产生活密切相关的“十三件实事”，以确保城市建设取得实效，尽快地让城市绿起来、亮起来、美起来、活起来，为人民群众创造一个空气清新、环境优美、舒适宜人的生产生活环境。

　　城市管理要组建“一支队伍”，打好“四个硬仗”。要坚持建设与管理并重、管理重于建设的指导思想，切实把城市管理拿在手上，管出成效。要进一步理顺城市管理体制，成立市政府城市管理行政执法局，强化综合执法。要继续引深“城市管理年”活动，深入开展城市环境综合整治，认真组织坚决打胜取缔马路市场、治理环境卫生、整顿交通秩序、规范户外广告四个硬仗，下大功夫治理脏、乱、差。要把泽州路、凤台街作为样板街，带动城市环境的进一步好转。要组织开展“做文明晋城人，建文明晋城市”活动，大力引导广大市民增强城市意识，提高文明素质，树立文明形象，大力营造热爱晋城、关心晋城、建设晋城的浓厚氛围，全方位提高城市文明程度，今年要进入省级文明城市行列。

　　(五)千方百计扩大就业，进一步完善社会保障体系

　　就业是民生之本，扩大就业是各级政府面临的重大而艰巨的任务。因此，我们必须把控制失业率和增加就业岗位作为宏观调控的重要指标来考核，继续实行就业、再就业目标责任制。要充分发挥非公有制经济和中小企业在促进就业方面的重要作用，引导失业人员和新增劳动力转变就业观念，加快形成劳动者自主择业、市场调节就业、政府促进就业的机制。

　　要进一步完善社会保障体系。继续做好“两个确保”工作，完善城镇职工基本养老保险制度，积极推进国有企业下岗职工基本生活保障向失业保险并轨，切实保障失业人员基本生活。继续提高城镇职工医疗保险覆盖面，积极推行农村新型合作医疗制度。进一步落实城乡居民最低生活保障制度。依法强化社会保障费的征收，凡不能保证社保资金按时足额缴纳的单位，一律不得建办公大楼、购买汽车、安排领导出国。

　　各位代表，目前，我市有城市低保对象3．5万多人，农村鳏寡孤独、老弱病残、天灾人祸等困难群体4．9万多人，保障这8．4万多人的基本生活需求是我们实践“三个代表”的具体体现，是各级政府义不容辞的责任，我们必须满腔热情，高度重视，认真对待，千方百计抓紧抓好。

　　(六)继续实施科教兴市战略，全面发展各项社会事业

　　保持经济与社会的协调发展，既是争先发展的出发点和落脚点，也是全面建设小康社会的本质要求。我们在狠抓经济建设的同时，必须把发展科技、教育和各项社会事业放在重要位置来抓。

　　要进一步加大科教兴市力度。引进和推广一批可以带动结构优化和产业升级的科技项目，把工业化与信息化结合起来，推动主导产业升级。全年组织实施各类重点科技项目50项。扶持科技信息网络建设，发展企业技术中心和研发中心，提高企业科技创新能力。一定要坚持教育优先发展的原则，加快城乡中小学布局调整，抓好中小学危房改造，加快建设教育信息网络，努力改善办学条件。要依法办好义务教育，积极发展幼儿教育和普通高中教育，壮大职业教育和成人教育，办好晋城职业技术学院。今年，要新建四中、五中综合教学楼和中原街小学。牢固树立人才是第一资源的观念，大力实施人才战略，营造引得进、留得住的人才环境，广开视野选人才，不拘一格用人才，努力在全社会形成尊重知识、尊重人才的良好风气。

　　要积极推动各项社会事业全面进步。继续加强计划生育工作，进一步稳定低生育水平，重视提高人口素质。要切实保护、合理开发和节约使用各种自然资源，大力加强城乡生态建设和环境治理工作。坚持正确的舆论导向，发展新闻出版、广播电视和文学艺术事业，深入开展群众性文化活动，引导扶持文化产业发展。加强社区卫生组织建设，发展农村卫生事业，强化食品、药品市场监管，做好计划免疫、疾病预防和地方病控制工作。提升竞技体育水平，繁荣群众体育活动，发展体育产业。抓好国防教育、民兵预备役和人防工作。深入开展“双拥”工作，搞好军民共建活动。进一步做好民政、宗教、外事、侨务、地震、档案、妇女、儿童、老龄、残疾人等各项工作，促进社会事业全面发展。

　　(七)推进社会主义精神文明和民主法制建设，营造有利于经济发展的社会环境

　　要切实加强精神文明建设。深入学习宣传党的十六大精神和“三个代表”重要思想，全面贯彻《公民道德建设实施纲要》，以诚信教育为重点，广泛进行社会公德、职业道德和家庭美德教育，进行世界观、人生观和价值观教育，弘扬爱国主义精神，不断提高人们的思想道德素质。按照创建一流文明城市的总体部署，广泛开展群众性精神文明创建活动。扎实抓好科技、文化、卫生“三下乡”和科教、文体、法律、卫生“四进社区”活动，倡导文明健康、积极向上的生活方式。

　　要切实加强民主法制建设。发展社会主义民主政治，建设社会主义政治文明。自觉接受人大及其常委会的依法监督，认真执行人大及其常委会的决定、决议，支持人大代表依法行使职权。加强与人民政协的协商，接受人民政协的民主监督。认真办理人大代表建议、议案和政协委员提案。广泛听取各民主党派和工商联的意见，发挥工会、共青团、妇联等人民团体的桥梁和纽带作用。继续推行村民自治，全面完成城市社区建设，进一步发扬基层民主。搞好普法宣传教育，提高公民法律素质，增强公务人员的法制观念和依法行政能力。

　　要切实加强社会治安综合治理。坚持“严打”方针，严厉打击各种刑事犯罪活动，严惩社会黑恶势力和团伙犯罪。认真落实社会治安综合治理措施，着力解决社会治安存在的突出问题，坚决查禁黄、赌、毒等社会丑恶现象。继续整顿和规范市场经济秩序，严厉打击制假售假等违法犯罪行为，严肃查处破坏市场经济秩序的大案要案。科技强警，从严治警，提高快速反应能力。正确处理人民内部矛盾，高度重视群众来信来访工作，认真解决好群众关心的热点、难点问题，妥善处理各种群体性事件，把各种不安定因素化解在基层和萌芽状态。

　　这里我要特别强调，我市以煤炭为主的产业格局，决定了必须高度重视安全生产，这是人命关天的大事，事关社会的稳定和政治的安定，责任重于泰山，丝毫不能懈怠。要大力营造“关爱生命，关注安全”的社会氛围，全方位落实安全生产责任制，加大对煤矿、道路交通等重点行业、重点领域、重点单位的安全防范和监督力度，坚决遏制重特大事故，切实减少一般性事故，为改革和发展营造良好的安全环境。

　　三、恪尽职守，开拓进取，确保今年各项目标的圆满实现

　　全面建设小康社会的新形势和新任务，对政府工作提出了更高的要求。各级政府必须把自身建设摆在突出位置，紧紧扭住“第一要务”，始终牢记“两个务必”，忠实实践“三个代表”，努力树立廉洁、勤政、务实、高效的政府形象。

　　一是要坚持与时俱进，进一步强化发展意识。面对当前全国各地竞相发展的局面，全市上下必须保持清醒头脑，百倍增强忧患意识。一定要自觉地把思想认识从那些不合时宜的观念、做法和体制的束缚中解放出来，从对马克思主义的错误的和教条式的理解中解放出来，从主观主义和形而上学的桎梏中解放出来，一切妨碍发展的思想观念都要坚决冲破，一切束缚发展的做法和规定都要坚决改变，一切影响发展的体制弊端都要坚决革除。我们虽然取得了一定的成绩，但这丝毫不能成为我们骄傲的理由，必须按照“两个务必”的要求，克服小富即安、小进即满的思想，抛弃夸夸其谈、坐而论道的做法，以更加开放的意识去审视存在的差距，以更加超前的理念去研究争先的思路，以更加扎实的干劲去落实发展的措施。

　　二是要坚持依法行政，进一步转变政府职能。要按照精简、统一、效能的原则，进一步理顺部门职能分工，推进行政管理体制改革。坚持依法行政，依法管理经济与社会事务，强化政府的经济调节、市场监管、社会管理和公共服务职能，致力于建设行为规范、运转协调、公正透明、廉洁高效的政府。建立健全领导、专家和群众相结合的民主决策机制，认真落实行政执法责任制和过错责任追究制，完善政务公开，实行“阳光作业”，接受社会监督。

　　三是要坚持求真务实，进一步转变工作作风。要大兴调查研究之风，深入实际，了解民意，集中民智，理清工作思路，把握工作重点。要下决心精文减会，减少应酬和繁杂的事务性活动，取消不必要的检查达标评比活动。领导干部到基层考察、调研，要轻车简从，简化接待，不搞迎送。要坚决反对形式主义和官僚主义，坚决克服弄虚作假、做表面文章等不良作风，坚决不搞劳民伤财、沽名钓誉的形象工程，集中精力办实事，求实效，促发展。

　　四是要坚持执政为民，进一步落实工作责任。要牢记为人民服务的宗旨，切实做到权为民所用，情为民所系，利为民所谋，对工作极端负责，对人民极端负责。坚持群众路线，尊重群众的首创精神，充分调动、发挥和保护全市上下争先发展的主动性和积极性。要进一步落实工作目标责任制，明确职责，各司其职，敢于负责。凡形成决策的事项，要不折不扣地定一件、办一件，办一件、成一件，取信于民。要公开曝光不落实的事，严肃处理不落实的人，确保政府各项工作目标的圆满实现。

　　五是要坚持廉洁自律，进一步发挥表率作用。要认真贯彻中央关于廉洁自律的各项规定，进一步增强反腐倡廉意识和拒腐防变能力。要严于律己，严格要求配偶、子女和身边工作人员，在公务活动中严禁权钱交易，严禁在资金安排、工程招标、政府采购和资源出让中以权谋私。加强制度建设，坚决纠正部门和行业不正之风，坚决惩治各种腐败行为，从源头上、机制上预防和治理腐败。

　　各位代表，我们正处在一个必须紧紧抓住并且可以大有作为的重要战略机遇期，我们面临的任务光荣而艰巨，我们肩负的责任神圣而重大，晋城能否发展得更快更好一些，是对我们这届政府的严峻考验。我们必须不辱使命，不负重托，勇挑历史重担，积极迎接

挑战。我们坚信，在以胡锦涛同志为总书记的党中央领导下，高举邓小平理论伟大旗帜，全面贯彻“三个代表”重要思想，有中共晋城市委的正确领导，有市人大的依法监督，有市政协的民主监督，有全市人民的共同努力，有社会各界的鼎力相助，有近年来经济发展打下的坚实基础，只要我们万众一心，乘势而上，任何艰难险阻都挡不住晋城人民奋勇前进的步伐，我们的目标一定要实现！我们的目标一定能够实现！
